# The Role of Therapeutic Drug Monitoring in Slow Response Extra-Pulmonary Tuberculosis Treatment: A Case Report

**DOI:** 10.7759/cureus.51301

**Published:** 2023-12-29

**Authors:** Abdulwahab M Aldrees, Mohammed H Alnajeim, Abdulrahman A Alomran, Abdulmohsen A Alshehri

**Affiliations:** 1 Internal Medicine/Infectious Diseases, King Khalid University Hospital, Riyadh, SAU; 2 Medicine, King Saud University Medical City, Riyadh, SAU

**Keywords:** slow responder, antituberculous drugs, treatment failure, tuberculous lymphadenitis, tuberculosis

## Abstract

Tuberculosis is caused by an infectious bacterium and it has significant morbidity and mortality rates globally. It mostly affects the lungs, but it can also spread to other parts of the body, like the lymph nodes (tuberculous lymphadenitis). The most common way to treat it is with the RIPE regimen, which includes rifampin, isoniazid, pyrazinamide, and ethambutol. The drugs can be slowly or rapidly metabolized, resulting in either increased toxicity or subtherapeutic drug levels. In this paper, we discuss the case of a slow responder who was treated with increased rifampin and isoniazid doses and improved clinically and biochemically. It's the first case of a slow responder diagnosed with tuberculous lymphadenitis reported in the Gulf region.

## Introduction

Tuberculosis (TB) continues to be a leading cause of illness and death globally. It is estimated that a staggering 1.7 billion people, roughly 22% of the world's population, are infected with *Mycobacterium tuberculosis* bacteria [1-2].

Despite being a preventable and curable disease, TB remains a major global health threat. According to the World Health Organization, an estimated 10.6 million people around the world fell ill with TB in 2022 [3]. This translates to an alarming 1.3 million deaths, with 167,000 of those coinfected with HIV [3].

While most cases of *Mycobacterium tuberculosis* infection manifest as lung disease, it is crucial to recognize that the infection can spread to other parts of the body, causing extrapulmonary TB [4]. Although less common, accounting for only about 10% to 15% of all cases, extrapulmonary TB presents a significant diagnostic challenge. This is due to the diverse range of organs and systems it can affect, including the lymph nodes (TB lymphadenitis), urinary tract and genital organs (genitourinary TB), and skin (cutaneous TB) [5].

Tuberculous lymphadenitis (TB LN) is the most frequent extrapulmonary manifestation of tuberculosis, with cervical lymphadenopathy emerging as its most common presentation. In recent studies, this form accounts for 63% to 77% of TB LN cases. However, diagnosing TB LN remains a significant challenge for healthcare professionals. This is due to the way it presents symptoms like other diseases, which makes it difficult to distinguish it definitively. Inconsistent findings in physical examinations and laboratory tests may not always provide conclusive evidence of TB LN. Accurate diagnosis frequently necessitates a tissue biopsy procedure [6-8].

Certain patients may experience treatment failure as a result of being slow responders to the anti-tubercular regimen. There are multiple reasons for this, including incorrect dosing, changes in metabolism, poor absorption, and interactions with other drugs, all of which can lead to low levels of the drug in the blood. Additionally, certain host variables like diabetes or HIV can also contribute to this [9]. Individuals may exhibit metabolic changes in response to the anti-tubercular medicine isoniazid. Individuals can exhibit either rapid or slow acetylation rates, which are determined by their genetic makeup. The medicine is deactivated by the enzyme N-acetyltransferase-2 [10]. The outcome is the administration of insufficient doses, leading to the eventual ineffectiveness of the tuberculosis treatment. Therapeutic drug monitoring (TDM) facilitates the identification of therapy failure. Understanding how to modify dosages based on a patient's drug elimination pathway is crucial for achieving optimal outcomes [11].

In this paper, we present the case of a 24-year-old Saudi male diagnosed with TB LN who failed to respond to the standard regimen doses. His therapeutic drug monitoring (TDM) panel showed low serum levels of rifampin and isoniazid. This is the first study done on such a case in Saudi Arabia.

## Case presentation

A 24-year-old male from the southern region with no medical or surgical background presented to the emergency department complaining of an on-and-off fever for 20 days. The fever ranged between 38.1 and 39 degrees Celsius. It was associated with night sweating, productive coughs with yellowish sputum, malaise, arthralgia, early satiety, and 9 kg of weight loss in 20 days. The patient also reported a history of visiting animal farms, including sheep and camels, and consuming unpasteurized milk regularly. In addition, the patient had a history of traveling to nonendemic TB areas, engaged in protected heterosexual intercourse, and was a long-term smoker. The rest of the patient’s history and systemic review were unremarkable. The patient first presented to a local hospital in his town and was given intravenous antibiotics and analgesics with no improvement, so he was instructed to visit a tertiary hospital for further examination.

On physical examination, he was febrile, conscious, alert, and oriented to time, place, and person. His weight was 70 kg, his BMI was 24.22, and he was hemodynamically stable and off oxygen. A full neurological, cardiac, respiratory, musculoskeletal, mucosal membrane, and joint space exam showed nothing unusual. However, there was a painless 1.2-cm right anterior cervical lymph node that could be felt, as well as hypopigmented spots and patches on his chest and neck and brown, scaly papules all over his neck that were later diagnosed to be tinea versicolor after a punch biopsy and pathological review.

**Figure 1:**
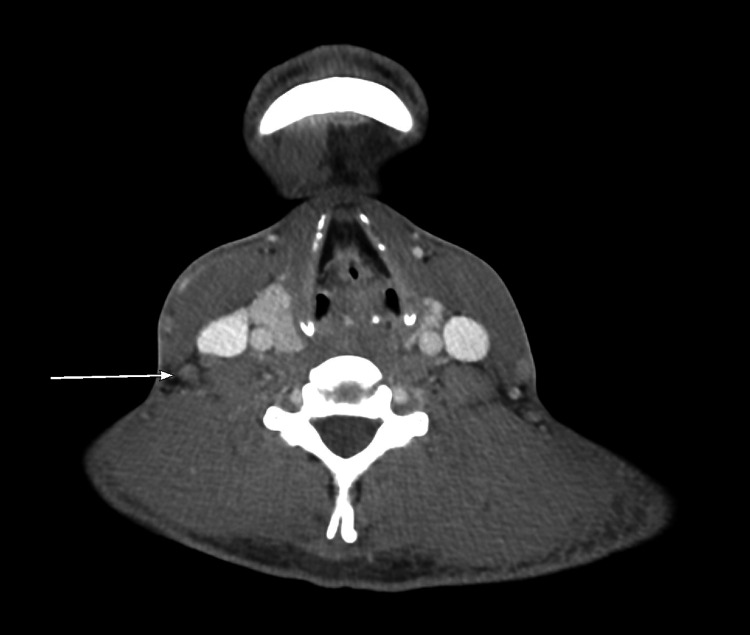
CT scan neck (axial view) at the time of admission View of right-side borderline-enlarged lower cervical and supraclavicular lymph nodes.

**Figure 2:**
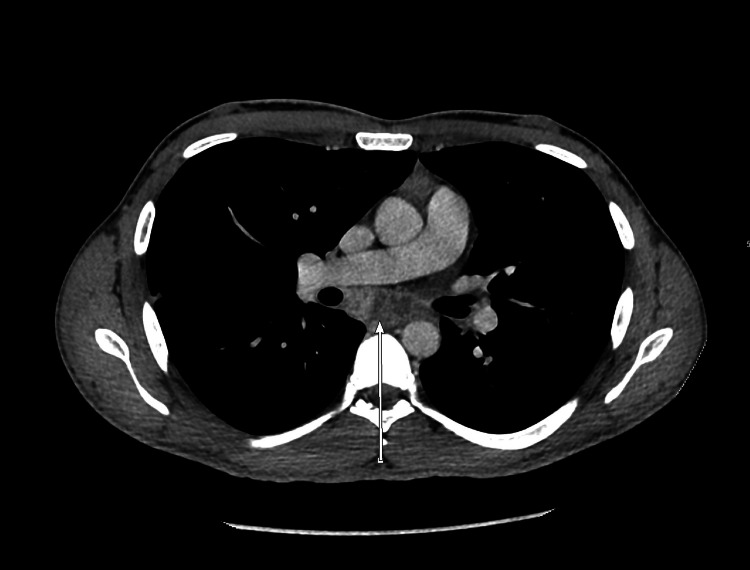
CT scan chest (axial view) at the time of admission The arrow shows the enlarged subcarinal lymph node (29 mm).

CT scans of the patient’s neck, chest, abdomen, and pelvis with contrast revealed enlarged mediastinal and cervical lymph nodes (Figures 1-2) were obtained. An endobronchial ultrasound-guided biopsy (EBUS) was done to collect cells from the subcarinal and precarinal lymph nodes. The pathology report was indeterminate, as it only showed diffuse, poorly preserved, and degenerative clusters with a focal monotonous population of lymphocytes. No well-defined granuloma was identified. Mediastinoscopy was suggested for further evaluation and the culture from the EBUS tissue sample was positive for *Mycobacterium tuberculosis*.

The diagnosis of TB LN that is sensitive to quadruple antitubercular medications (rifampin, isoniazid, pyrazinamide, and ethambutol) was established, and a treatment plan was initiated with standard doses: rifampin 300 mg, isoniazid 150 mg, pyridoxine 40 mg/day, pyrazinamide 1500 mg, and ethambutol 1200 mg. The two latter drugs were stopped after two months. The patient initially noticed an improvement in night sweats, fatigue, and fever, and no obvious side effects were reported.

**Figure 3:**
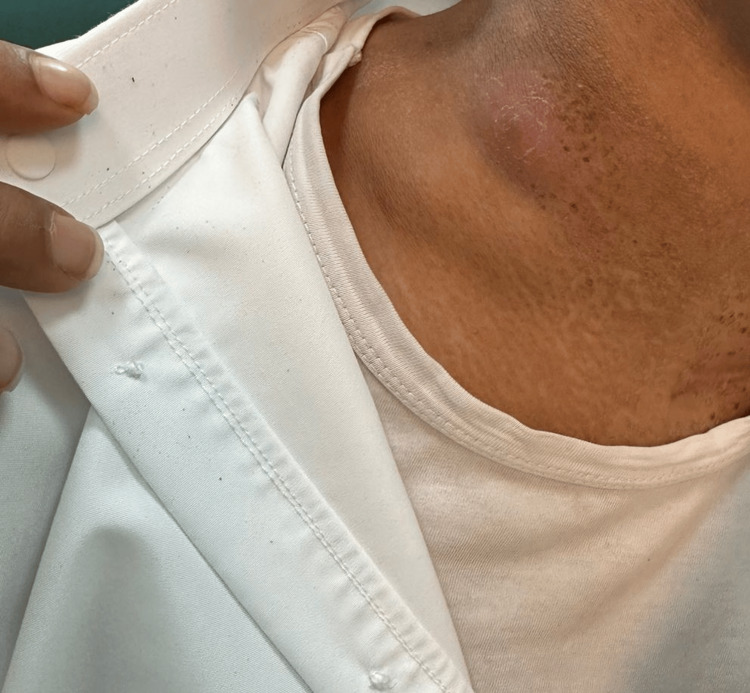
The patient's neck after about five months of treatment The image shows a 5*4 cm non-tender fluctuant nodule.

Four and half months into treatment, the patient presented to the emergency department complaining of an on-and-off fever, productive cough, and painless right-sided cervical swelling with oozing pus. He was admitted for further examination. The patient’s acid-fast bacteria (AFB) sputum smear and polymerase chain reaction (PCR) test came back positive for three samples (Figure 3).

**Figure 4:**
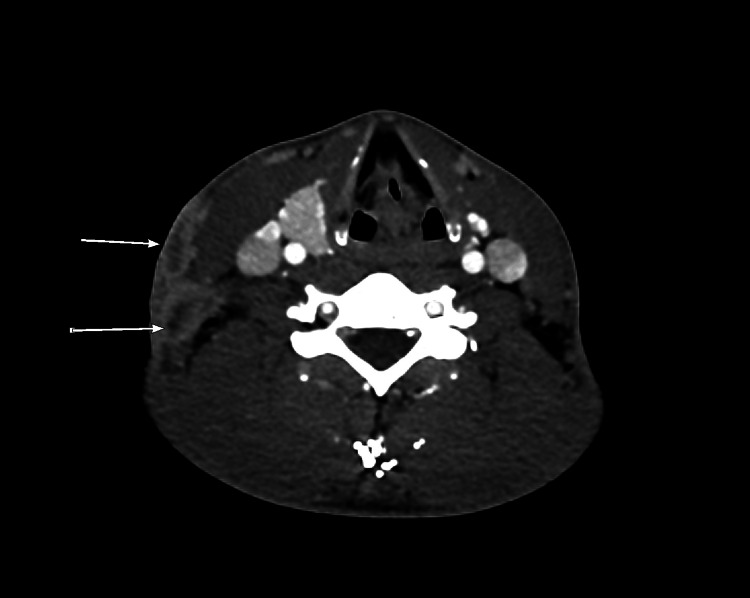
CT scan neck (axial) after about five months of treatment Interval development of left lower cervical, left supraclavicular, and left paratracheal necrotic lymph nodes extending down into the mediastinum.

**Figure 5:**
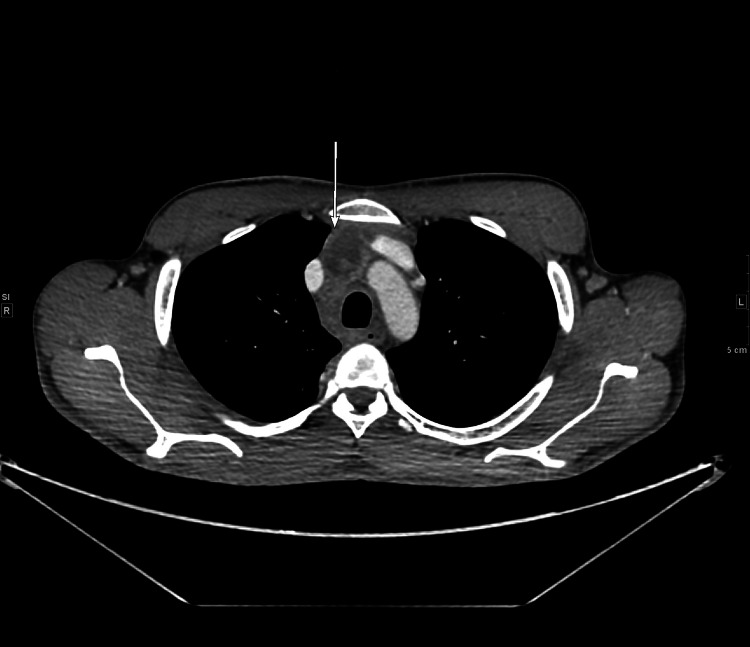
CT scan chest (axial view) after about five months of treatment Newly enlarged lymph node shows the progression of the disease and failure of the previous standard regimen. It is a 25x30 mm prevascular necrotic lymph node.

**Figure 6:**
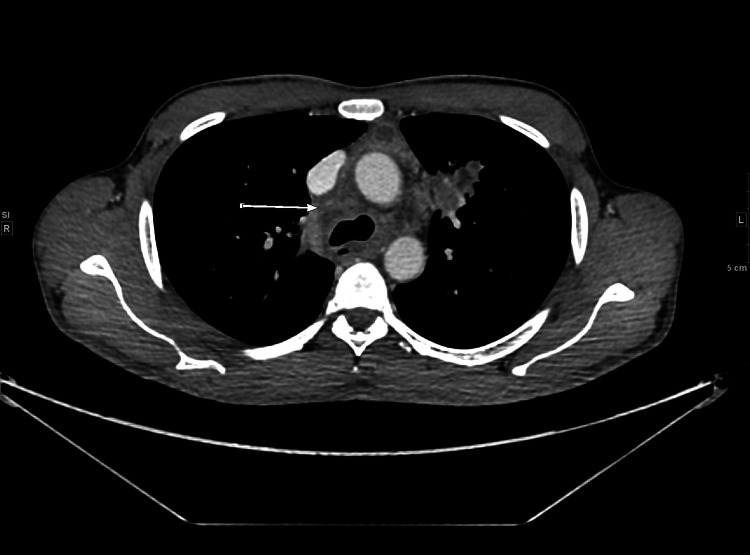
CT scan chest (axial view) showing interval increase in right paratracheal lymph node size This CT scan image shows that the paratracheal lymph node has increased to 18 mm in size from 9 mm in previous images when the patient was first treated.

A CT scan of his chest and neck showed a progression of lymph node enlargement in the neck and multiple pulmonary nodules (Figures 4-6). The patient was again started on quadruple antitubercular medications with standard doses, plus moxifloxacin 400 mg. Two weeks later, the repeated AFB sputum smear was negative for three samples. Drug levels were sent, with the results showing the following: rifampin 4.36 mg/L at two hours and not detected at six hours; isoniazid with a slight accumulation of acetyl-isoniazid at six hours; ethambutol peak 1.7 mg/L (low); and pyrazinamide 175 mg/L at two hours (high) and 16.6 mg/L at six hours. It was suspected that the patient was a slow responder, so dosages were adjusted (rifampin 900 orally once a day (PO OD) and isoniazid 450 PO OD). Pyrazinamide, ethambutol, and moxifloxacin were discontinued after two months. The expected duration of treatment was six to nine months, according to improvement. Two months into the treatment, the patient reported improvement in his symptoms, with a weight increase from 57 kg to 60 kg. At six months, the patient had completely improved clinically and biochemically. Two months later, there was a follow-up to ensure all labs were normal, at which point he was discharged. Monthly liver enzymes were regularly followed and remained at the upper normal limit throughout the course of treatment.

## Discussion

Treatment for tuberculosis can be difficult, and precisely diagnosing the reason for treatment failure is critical for making future decisions and reducing disease morbidity and mortality. Because the patient satisfies the criteria for TB treatment failure, therapeutic drug monitoring for correct treatment was advised [12,13].

Therapeutic drug monitoring (TDM) never serves as a replacement for Directly Observed Therapy (DOT) or for good clinical judgment. Nonetheless, TDM is a helpful tool in a range of clinical scenarios, such as individuals who have a delayed response or who are at high risk of treatment failure. By enabling the achievement of the intended serum concentrations, TDM raises the likelihood of favorable bacteriological and clinical results. A number of TB medications exhibit significant inter-individual variability. Complicated drug-drug interactions can also be resolved by TDM before the patient develops toxicity, failure, or relapse. TDM is an extremely effective ally in the management of intricate clinical problems in several respects. To further identify therapeutic concentrations, more research is required, particularly for second-line TB medications [13].

The patient's TDM revealed low drug levels of both rifampin and isoniazid at two hours of measured concentration, implying that the treatment was unsuccessful. Prahl et al. [14] from Denmark presented their article "Clinical Significance of 2-Hour Plasma Concentrations of First-Line Tuberculosis Drugs" at the 2013 Interscience Conference on Antimicrobial Agents and Chemotherapy (ICAAC). Two hours following the treatment, 35 people supplied a blood sample. In 25 of 35 patients (71%), the amount of isoniazid in the blood was below the normal range. The same was true for rifampicin in 19 of 33 patients (58%), ethambutol in 13 of 28 patients (46%), and pyrazinamide in 3 of 29 patients (10%). Patients with low isoniazid, rifampicin, or both levels had a higher rate of treatment failure [14].

The patient exhibited improvement after six months of dose adjustment of isoniazid and rifampin, as suggested by the trials [15]. However, it is important to note that these trials were conducted on a distinct population and specifically targeted pulmonary tuberculosis. By reporting a slow response to TB LN in Saudi Arabia, researchers might get valuable insights into the appropriate dosage adjustments, treatment outcomes, and side effects specific to TB LN, as opposed to the more prevalent pulmonary tuberculosis [15]. Additionally, it has the capacity to address the lack of epidemiological data on individuals who respond slowly in the Gulf region.

## Conclusions

When individuals do not respond well to treatment within the initial months, it is important to consider the possibility of slow responders. Diagnosis of slow responders primarily relies on clinical judgment and DOT. However, TDM plays a crucial role in ensuring accurate diagnosis and aiding in drug therapy. Consequently, the antitubercular treatment regimen for slow responders should differ from that of the general population. This can be achieved by increasing the doses of both rifampin and isoniazid.
